# Probiotics and Postbiotics as an Alternative to Antibiotics: An Emphasis on Pigs

**DOI:** 10.3390/pathogens12070874

**Published:** 2023-06-26

**Authors:** Md. Sekendar Ali, Eon-Bee Lee, Walter H. Hsu, Kyoungho Suk, Syed Al Jawad Sayem, H. M. Arif Ullah, Seung-Jin Lee, Seung-Chun Park

**Affiliations:** 1 Department of Biomedical Science and Department of Pharmacology, School of Medicine, Brain Science and Engineering Institute, Kyungpook National University, Daegu 41944, Republic of Korea; alipharm@iiuc.ac.bd (M.S.A.); ksuk@knu.ac.kr (K.S.); 2 Laboratory of Veterinary Pharmacokinetics and Pharmacodynamics, College of Veterinary Medicine, Kyungpook National University, Daegu 41566, Republic of Korea; eonbee@gmail.com (E.-B.L.); aljawadsayem@gmail.com (S.A.J.S.); 3 Department of Pharmacy, International Islamic University Chittagong, Kumira, Chittagong 4318, Bangladesh; 4 Department of Biomedical Sciences, Iowa State University, Ames, IA 50014, USA; whsu@iastate.edu; 5 Department of Neurobiology, University of Utah, Salt Lake City, UT 84112, USA; hmarif.ullah@neuro.utah.edu; 6 Development and Reproductive Toxicology Research Group, Korea Institute of Toxicology, Daejeon 34114, Republic of Korea; 7 Cardiovascular Research Institute, Kyungpook National University, Daegu 41566, Republic of Korea

**Keywords:** probiotics, postbiotics, antibiotics, pigs, piglets

## Abstract

Probiotics are being used as feed/food supplements as an alternative to antibiotics. It has been demonstrated that probiotics provide several health benefits, including preventing diarrhea, irritable bowel syndrome, and immunomodulation. Alongside probiotic bacteria-fermented foods, the different structural components, such as lipoteichoic acids, teichoic acids, peptidoglycans, and surface-layer proteins, offer several advantages. Probiotics can produce different antimicrobial components, enzymes, peptides, vitamins, and exopolysaccharides. Besides live probiotics, there has been growing interest in consuming inactivated probiotics in farm animals, including pigs. Several reports have shown that live and killed probiotics can boost immunity, modulate intestinal microbiota, improve feed efficiency and growth performance, and decrease the incidence of diarrhea, positioning them as an interesting strategy as a potential feed supplement for pigs. Therefore, effective selection and approach to the use of probiotics might provide essential features of using probiotics as an important functional feed for pigs. This review aimed to systematically investigate the potential effects of lactic acid bacteria in their live and inactivated forms on pigs.

## 1. Introduction

One of the greatest scientific breakthroughs of the 20th century was the development of antibiotics, which allowed the control of numerous diseases. However, due to their overuse/misuse, new dangers, such as antibiotic resistance have emerged in human and veterinary medicine [1]. Studies have shown that consuming meat and meat products from antibiotic-fed pigs is indisputably bad for consumers’ health because even low concentrations of antibiotics can create problems regardless of the length of time [2,3]. In other words, antibiotics in pigs should only be used therapeutically and not to improve pigs’ health conditions or promote growth and development. Therefore, it is not acceptable to maximize the fattening of pigs using antibiotics for only financial reasons [4]. In order to meet the needs of society worldwide, agriculture has evolved, and intensive pig production has been introduced. An unexpected side effect of intense pig breeding is that it makes farms more crowded and encourages the spread of many diseases [5]. Overcrowded pig production environments make it much easier for pathogens such as parasites, viruses, fungi, and bacteria to transmit to humans through zoonotic transmission [6].

The pig industry has had excellent development and strong demand, which has led to a steady rise in production in recent years. There were 677.6 million pigs in the world in 2020, with China being the leading producer and providing more than half of the total pig population [7]. The remaining portion of the world’s total pork production is attributed mostly to the EU, the USA, Brazil, and Russia. The EU produced 22.8 million tons of pigs [8], and the USA produced 4.3 million tons [9]. In contrast, Korea has reached over 2.3 million tons of pork production [10].

According to estimations, livestock use approximately twice as many antibiotics as humans do worldwide [11]. For instance, each kilogram of meat produced in the US requires the usage of around 300 milligrams of antibiotics [12]. These drugs are used for treatment as well as to prevent infections. They are utilized to promote animal growth in many countries. People have been infected with bacterium strains resistant to antibiotics since many farmers started providing antibiotics to livestock in the late 1940s [13]. In an effort to stop this risky practice, the EU banned the use of antibiotics for livestock growth in 2006 [14]. In the USA, Food and Drug Administration recommended farmers stop the use of antibiotics voluntarily [15]. However, the use of probiotics in livestock production has been increasing as an antibiotic alternative to preventing infection by boosting immunity.

Probiotics can help maintain the balance of intestinal microbes, and they have an antibiotic-like impact on pathogenic microorganisms in pigs [16]. Probiotics are live microorganisms that provide health benefits upon consumption, usually by restoring or improving the gut flora. According to the World Health Organization’s (WHO) definition, probiotics are non-pathogenic living microorganisms that provide a health advantage to the host when administered in optimum amounts [17]. Probiotics, mostly *Lactobacillus* or *Bifidobacterium* species, are derived from the intestinal microbiota of animals, humans, and dairy products [18]. Probiotics also include yeasts such as *Saccharomyces* and bacteria of the genera *Bacillus*, *Enterococcus*, and *Streptococcus* [19]. Probiotics have the potential to act against various infectious agents. Lactic acid probiotic bacteria, including *Lactobacillus*, *Streptococcus*, and *Bifidobacteria*, have been demonstrated to suppress *Helicobacter pylori*, *Salmonella*, and *E. coli* [20]. They can fight against pathogens by producing different antimicrobial components, lowering pH, competing with pathogens for adhesion and nutrients in the gut, and suppressing pathogenic bacterial growth via direct coaggregation with the bacteria [21,22]. Probiotics also have antiviral and antifungal properties. Previous studies revealed that lactic acid bacteria (LAB) as probiotics such as *Lactobacillus* and *Bifidobacteria* could inhibit the fungus and virus from absorption into the intestine by steric hindrance, boosting the barrier integrity of the mucosa, or by competition for specific target receptors [23,24]. Probiotics can affect the immune responses mediated by different types of immune cells, such as T and B lymphocytes, macrophages, and natural killer (NK) cells [25]. The innate immune system has been demonstrated to be linked to probiotics with the expression of cytokines by antigen-presenting cells, activation of NK cells, and induction of type 1 helper T cell response [26]. The acquired immunity can also be boosted with probiotics by enhancing the production of immunoglobulins by lymphocytes [27].

Notwithstanding the widespread use of probiotic supplementation in various conditions, there has been concern regarding the likelihood of unwanted effects associated with live probiotics, especially in children and adults suffering from diseases [28]. One of the biggest concerns of using live probiotics is that they may be transported from the gut to blood vessels and nearby draining tissues, resulting in bacteremia, specifically in pediatrics, immunocompromised, and seriously ill patients [29]. The other issues associated with using live probiotics include the possibility of probiotic strains transmitting or acquiring antibiotic-resistant genes via gene transfer in the human gastrointestinal tract (GIT), harmful metabolic activities, and overstimulation in vulnerable individuals [30]. Live probiotics may create a permanent colony in neonates, preventing normal colonization of other bacteria or normal microbiota in the GIT and altering the normal development of the immune system [31]. Therefore, other agents, such as postbiotics, are being considered in response to these issues.

Postbiotics have gained much attention from the scientific community for their potential applications in the functional food and pharmaceutical sectors. Although verbal inconsistencies are present in defining postbiotics, there is a standard consensus definition for facilitating the advancement of this field. According to the definition provided by the International Scientific Association for Probiotics and Prebiotics (ISAPP), “Postbiotic is a preparation of non-living microorganisms or their constituents that provides a health advantage to the host” [32]. Based on this definition, postbiotics are inactivated bacteria in whole or part, with or without metabolic byproducts. The published research on these preparations uses a number of distinct terminology terms, including inanimated probiotics, tyndallized probiotics, heat-killed probiotics, cell lysates, parabiotics, ghost probiotics, and postbiotics [32]. Many postbiotic preparations also contain microbe-produced components such as metabolites, peptides, enzymes, proteins, vitamins, and exopolysaccharides (EPSs), which may contribute to the overall health benefits conferred by a postbiotic [33]. They are used because research suggests that probiotic effects are mediated by specific effector molecules by interacting with host cells. Postbiotics offer more advantages than live probiotics; for instance, (a) live probiotics may have trouble adhering to the gut mucosa because of the mucous layer that limits the direct contact between the gut mucosal layers and bacteria. However, postbiotics can pass through the mucous layer quickly [34]. (b) There are no risks of infection due to bacterial translocation from the intestinal lumen to the blood in susceptible and immunocompromised individuals. Therefore, there is no possibility of acquiring and transferring antibiotic resistance genes. (c) It is convenient to maintain the dose and easy to transport, standardize, and store postbiotics.

Different active cellular components of probiotics produce various immune responses. Previous studies have demonstrated that probiotics’ cell-free supernatants [35], EPSs [36], peptidoglycans [37], teichoic acid and lipoteichoic acid [38], and active metabolites [39] modulate immunity through the stimulation of innate and adaptive responses.

Postbiotics, also called inactivated probiotics, are non-viable bacteria upon oral administration in adequate amounts to bestow a benefit on the animal or human consumers. According to the latest scientific literature, postbiotics are often defined as “Non-viable, dead, or inactivated probiotic microbial cells (ruptured or intact cells containing cell components) or their crude extracts” [40]. Postbiotics, therefore, are a complex mixture of beneficially secreted or metabolic probiotic compounds found in the supernatant, such as organic acids, amino acids, short-chain fatty acids (SCFAs), vitamins, peptides, enzymes, and secreted proteins [41]. The postbiotics form of probiotic bacteria can also be obtained by applying optimal heat, ultraviolet (UV) light, and sonication. Thus producing killed bacteria may be a safer alternative than live probiotics in susceptible groups, such as neonates, and may potentially play a role in the treatment of gastrointestinal diseases as well as immune disorders [42,43]. In their inactivated form, different strains of bacteria, such as *Lactobacillus* and *Bifidobacteria* can have beneficial effects in terms of immunoregulation [44,45]. Numerous studies showed that beside dead cells, culture supernatants, cell fractions, and metabolites of probiotic bacteria could have biological activities [46,47]. Although there are various modes of killing bacteria, such as sonication, mechanical pressure, and UV treatment, heat inactivation is widely used to obtain significant biological effects. Different microbiological components, including EPS [48], teichoic and lipoteichoic acids [49], lipopolysaccharides (LPSs) [50], peptidoglycans [51], cell-free supernatants [46], and metabolites [34] show immunomodulatory properties by stimulating innate immunity [46], adaptive immunity [52], and their effects on the integrity of the intestinal epithelial cell membrane [53]. These compounds usually act on different signal transduction receptors, including Toll-like receptors (TLRs) in the dendritic cells, intestinal epithelial cells, and many other immunological cells [54]. This review aimed to focus on probiotics and postbiotics and their role in immunomodulation, as well as their potential use in the benefit of pork production.

## 2. Probiotics as a Source of Functional Components

### 2.1. Antimicrobial Agents

The antimicrobial properties of probiotic microorganisms are diverse. *Lactobacillus* strains produce both nonspecific and specific antibacterial compounds and toxins, such as bacteriocins and bacteriocin-like components [55]. *Lactobacillus* produces bacteriocins, which have potent antibacterial properties and act in a bactericidal or bacteriostatic manner against various food pathogens [56]. Bacteriocin production has always been regarded as a significant character in the selection of probiotic strains [57]. Bacteriocin synthesis can also contribute to intestinal *Lactobacillus* probiotic activity and, in some cases, may be directly responsible for it in terms of either beneficially altering the gut microbiota or suppressing some gastrointestinal pathogenic bacteria [58]. As a result, bacteriocins generated from probiotic *Lactobacillus* can be used in a wide range of applications, including the food industry and medicine, and are often used in combination with other treatments to improve their efficacy in humans and animals [59]. Different SCFAs, such as lactic, formic, butyric, and propionic acids, are produced during carbohydrate metabolism in the presence of the probiotic *Lactobacillus* [60]. SCFAs have recently gained popularity as a treatment option for irritable bowel syndrome and colorectal cancer because they can reduce inflammation and inhibit the growth of malignant cells [61]. Previous studies showed that the administration of *L. reuteri* to patients with irritable bowel syndrome resulted in an increase in butyrate and acetate as well as a reduction in pro-inflammatory cytokines [62]. Moreover, SCFAs can improve Caco-2 cell’s transpermeability by expressing the tight junction proteins, suggesting that SCFAs may have an impact on modulating the intestinal barrier functions and may be pertinent for specific consumers in various stages of life [63]. 

### 2.2. Vitamins

It is commonly known that mammals are unable to synthesize most vitamins. However, some intestinal bacteria such as *Lactobacillus* can produce vitamins such as folate, vitamin B12, and riboflavin [64]. Gut microbiota have been identified as a source of such vitamins, and these vitamins have been described because of the fermentation of *Lactobacillus* in cheese, yoghurt, and other fermented products. These vitamins play an important role in essential functions such as antioxidants and cell metabolism. A previous study showed that different *Lactobacillus* spp. such as *L. lactic*, *L. plantarum*, and *L. bulgaris* are capable of producing folate [65]. As vitamin B12 cannot be produced by mammals, it must be obtained from a foreign source or intestinal microbiota. It has been found that some *Lactobacillus* spp. can synthesize this vitamin. A well-known probiotic *L. reuteri* demonstrated the ability to produce vitamin B12 in animals [66]. Another essential vitamin, riboflavin, which is vital in cellular metabolism, can be produced by *Lactobacillus* strains isolated from wheat flour used to prepare bread and pasta to improve them with riboflavin production [67].

### 2.3. Peptides

The proteolytic system of probiotics is capable of releasing peptide molecules from different foods. Judicious selection of probiotics is efficient in producing bioactive peptides from milk products. Via fermentation of milk with LAB such as *L. rhamnosus* GG, hydrolyzed caseins are produced, which are capable of lymphocyte proliferation [68], phagocytosis and macrophage maturation [69], and splenic lymphocyte proliferation [70]. The other peptides, such as beta-lactoglobulin, which can be hydrolyzed by *Lactobacillus paracasei*, are capable of modulating the production of interleukin (IL)-10, IL-4, and interferon (INF)-γ [71]. *Lactobacillus helveticus* proteolytic products possess immunoprotective properties against different bacterial infections [72].

### 2.4. Biosurfactants

Biosurfactants (BSs) are a class of various polymeric compounds that are secreted extracellularly or attached to cell walls and formed during the late log or early stationary phase of an organism’s growth cycle. There are a number of BSs, such as glycolipids, phospholipids, lipopeptides, polysaccharide–protein complexes, neutral lipids, and free fatty acids, that have been well recognized [73]. The BSs are amphiphilic in nature, assisting in the disruption of already-formed biofilms or delaying the onset of biofilm development by pathogenic microbes. Additionally, the foaming, wetting, and emulsification characteristics make it difficult for the pathogenic bacteria to attach, colonize, and communicate in the biofilms [74]. Properties such as anti-adhesion, emulsion stabilization, anti-biofilm, antimicrobial, and immunomodulatory abilities of BSs have been exploited as far as the food, biomedical, and pharmaceutical application sectors [75].

### 2.5. EPSs

Probiotic LAB have attracted the attention of researchers due to their capacity to produce EPSs, mainly because of their immunomodulatory properties. Many *Lactobacillus* strains yield EPSs, which stay attached to the outer cell walls and have immunomodulation capabilities. EPSs produced by *L. rhamnos* GG can block the TLR on macrophages and attenuate the pro-inflammatory cytokines by regulating NF-κB pathways and thus ameliorates the cancer progress [76]. Evidence suggests that the EPSs secreted by *L. acidophilus* DSMZ 20079 improve immunological functions by modulating the levels of IL-2, IL-8, and tumor necrotic factor (TNF)-α [77]. *Lactobacillus rhamnosus* RW-9595M produces EPSs which have the ability to stimulate IL-6, TNF-α, and IL-12 in humans and cultured immunocompetent cells of mice and IFN-γ in splenocytes of mice, suggesting the probability of improving the immunity of lactic acid bacterial EPSs in individuals reactive to this stimuli [78]. *L. paracasei* NTU 101 and *L. paracasei* NTU 102 produce EPSs that modulate the expression of IL-6, IL-1β, and TNF-α, and enhance the phagocytosis activities of RAW cells [79].

### 2.6. Enzymes

Probiotics have the capacity to produce enzymes that have anti-inflammatory activities. *Streptococcus thermophillus* can produce catalase or superoxide dismutase, showing anti-inflammatory actions in mice [80]. Under other circumstances, these bacteria could be used to prepare enzyme extracts that are capable of functioning in the fermentation environment [81]. The enzymatic activity of *Lactobacillus* isolated from fermented products such as yoghurt and cheese has been extensively studied [82]. The peptidase and dipeptidase produced by *L. lactis* can be used to produce cheese and bread [83].

## 3. Source of Postbiotics and Their Potential Benefits

The therapeutic effects of postbiotics including different cell wall components of probiotics have been proven. In the case of using a product containing probiotic strains, identification and selection of key components are necessary, along with the assurance of maintaining their activities. Among many other cell wall components of probiotics, the major cell wall components of *Lactobacillus* are lipoteichoic acid, peptidoglycan, and surface-layer proteins, which have been associated with immunomodulatory activities.

### 3.1. Lipoteichoic Acid

A previous study showed that the lipoteichoic acids of *L. plantarum* had the potential to induce IL-12, thereby stimulating innate immunity in the culture of splenic dendrites of mice [84]. In a study of pig epithelial cell lines, lipoteichoic acid of *L. plantarum* was found to possess anti-inflammatory activity by downregulating the expression of IL-8, showing the potential of this component to modulate the immune response [49].

### 3.2. Peptidoglycans

Peptidoglycans obtained from different *Lactobacillus* species have shown the capacity to modulate the expression of pro-inflammatory cytokines in LPS-treated murine macrophage cell lines [85]. Peptidoglycan from *L. rhamnosus* demonstrated to boost the innate immunity in *S. pneumoniae*-infected immunocompromised mice [86]. Furthermore, nasal injections of this molecule improved innate immunity and also elicited respiratory and adaptive immune effects in humans [87].

### 3.3. Surface-Layer Proteins

Surface-layer (S-layer) proteins are the combination of proteins and glycoproteins that cover the cell surface found in various species of probiotic bacteria, forming porous and symmetric layers to completely wrap the cell surface [88]. They are present on the cell surface of *Lactobacillus*. For instance, the ability of the S-layer protein of *L. acidophilus* to attach to dendritic cells produces an immunoregulatory phenotype (Treg) and enhances mucosal homeostasis which has been linked to the capacity of probiotic bacteria to bind with dendritic cells to stimulate an immune response [89].

### 3.4. Cell-Free Supernatants and Soluble Factors

Cell-free supernatant and different soluble factors in probiotic culture media, such as metabolites and other released products, can pass through the mucous layer and interact with the mucosal immune cells in the intestinal monolayer [90]. *Lactobacillus* metabolites possess antioxidant and anti-inflammatory properties, acting initially on gut mucosal cells and later on immunity, depending on the type of probiotic strain [34]. An in vitro model of immune cell lines has been shown to reduce pro-inflammatory mediators after being exposed to *Lactobacillus*-secreted products [34]. In a previous study, various probiotic strains, including *L. acidophilus*, *L. reuteri*, *L. plantarum*, *L. casei*, and *L. rhamnosus*, showed their immunomodulatory activities in peripheral blood mononuclear cells (PBMCs), which were mediated by cell-free components and metabolites of these bacteria [91]. In another study, it was revealed that cell-free supernatants of *L. rhamnosus and L. casei* possessing metabolites were capable of reducing the colon cancer cell invasion [92].

## 4. Potential Immunomodulation by Live Probiotics

**Figure 1:**
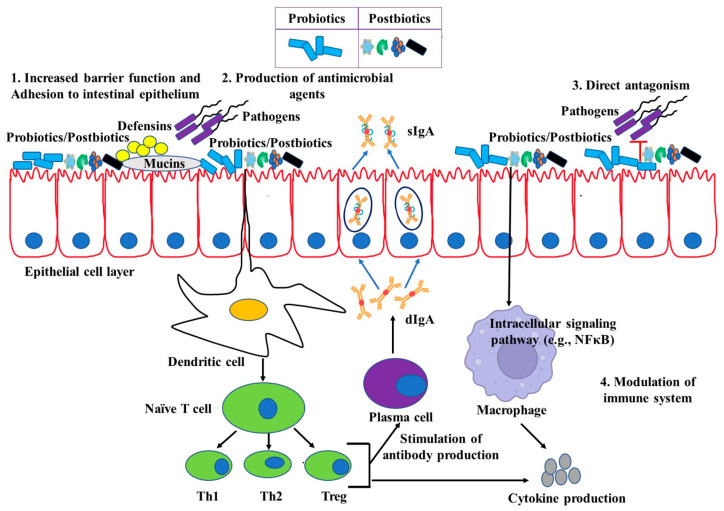
Mechanisms of action of probiotics and postbiotics.

There are numerous investigations demonstrating that live probiotics show immunoregulatory effects and defend the body from potential infections [93]. Probiotic *Lactobacillus* can modulate cell-mediated immunity, which is mediated by immune cells such as macrophages, NK cells, T-lymphocytes, and the expression of cytokines [26]. Probiotics present anti-inflammatory reactions by modulating various signaling pathways [94]. Diverse immunomodulatory and immunoprotective impacts on the intestinal epithelial cells have been delineated by probiotics; for instance, they can improve the epithelial barrier functions of the intestine [95,96]; develop waning barrier dysfunction [97]; or modulate the intestinal inflammatory reactions by ameliorating T-regulatory reactions, which is significant for controlling chronic inflammatory disorders [98]. It has been demonstrated that probiotic *Lactobacilli* can inhibit the production of LPS-induced pro-inflammatory cytokines TNF-α and IL-6 in intestinal cells [99,100]. The intestinal epithelium is one of the major protective layers, comprising the mucous layer, secretory immunoglobulin A (sIgA), antimicrobial peptides, and tight junction adhesion molecules [95]. The probiotic can reinforce the epithelial integrity and protect from infectious pathogens (Figure 1). Moreover, probiotic bacteria can prevent inflammatory bowel syndrome, which is caused by cytokines, damaging the intestinal epithelium [101]. A few probiotic bacteria can hinder pathogens in penetrating the intestinal barrier by stimulating the formation of Goblet cells’ mucin granules, extending the mucous membranes, regulating intestinal permeability, and expanding the apical tight junction’s integrity [102].

## 5. Beneficial Effects of Probiotics on Pigs

Weaning the piglets from the sow in the fourth week is a crucial stage in breeding. Pigs are extremely sensitive to alterations in their living surroundings. The gut flora becomes unstable during this extremely stressful phase for piglets. There are more deaths during this time due to diarrhea and gastrointestinal problems [103,104]. *Salmonella*, *E. coli*, and *Clostridium perfringens* are the most frequently observed organisms that cause intestinal diseases and damage intestinal villi. During infection, there is an increase in the permeability of fluids to the intestinal lumen, and diarrhea occurs. Antibiotics are used to affect the gut flora and lessen the incidence of diarrhea during weaning. Pathogenic bacteria that are resistant to the common antibiotics used in pig farming have emerged as a result of antibiotic overuse and/or misuse. In addition, research on the use of LAB to enhance gut microbiota was initiated in the context of pig farms [105]. It has been demonstrated that probiotic bacteria isolated from pigs produce antibacterial substances which are active against pathogens [106]. The time-kill assay was used to evaluate the activity of *L. reuteri* PSC102 against enterotoxigenic *E. coli* (ETEC) strains (KVCC1423, KVCC0543, and KVCC0306) [107]. Moreover, the culture supernatant of *L. reuteri* PSC102 showed antibacterial activity against these pathogenic bacteria as assessed by the disk-diffusion method [107]. In another study, Wang et al. [108] showed that probiotic LAB isolated from pigs demonstrated antibacterial activity against *Staphylococcus aureus*, *Enterococcus faecalis*, *Listeria monocytogenes*, and *Staphylococcus* enterica as assessed by the agar well-diffusion method. These investigations provided evidence that some strains of LAB, such as *Lactobacillus acidophilus*, *Lactobacillus salivarius*, and *Lactobacillus delbrueckii*, isolated from pigs’ digestive tracts can prevent the growth of potential pathogens by producing organic acids along with bacteriocin-like proteins [106]. In an experiment, immunobiotic soy milk based on okara fermented with *L. delbrueckii* was fed to piglets. The quantity of *Lactobacillus* and *Lactococcus* increased immunity, and favorable intestinal flora also developed. These piglets demonstrated greater development and meat quality [109]. Additionally, it has been shown that using *L. delebureckii* as a dietary supplement enhanced piglets’ gut integrity and structure [110]. Weaned piglets treated with *B. licheniformis* yielded similar outcomes. The expression of antimicrobial peptides in the ileum and intestinal permeability were increased, and nutrients were more easily digested [111]. In a different experiment, *L. salivarius* MP100 was isolated from sow’s milk and administered to pregnant sows and piglets. The MP100 demonstrated antibacterial activity against different pathogenic bacteria such as *S. typhimurium*, *E. faecalis*, *S. aureus*, *E. coli*, *Streptococcus suis*, and *Klebsiella pneumoniae*. An improvement in the gut’s microbiome and biochemistry was seen after using probiotics [112]. The bacteriocin gassericin A is produced by *Lactobacillus gasseri,* which has the ability to adhere to the host’s intestinal epithelium and render it resistant to diarrhea in weaned piglets [113]. Gassericin A works by binding with keratin in the plasma membrane of the intestinal epithelium to boost fluid absorption from the intestine, thereby reducing its secretion. A previous study showed that oral administration of fecal microbiota obtained from healthy pigs to piglets suffering from diarrhea reduced the incidence of diarrhea [114]. This treatment boosted the immunity of the piglets and reduced stress-induced diarrhea. For the prevention of diarrhea in piglets under increasing stress, *L. gasseri* can be used instead of antibiotics [115]. In one study, feeding *L. salivarius* to nursing piglets early in life led to an increase in *Lactobacillus* and a decrease in *Bacteroides* and *Fibrobacter* in the digestive system [116]. Additionally, there was a decrease in the frequency of diarrhea during the most stressful part of weaning. Therefore, supplementation with *L. salivarius* can increase growth efficiency by increasing the body mass index. Moreover, this strain also decreases the amount of pathogenic bacteria (*Clostridium* spp.) in the feces [117]. 

Different ways of administration, such as the encapsulated form of LAB given to pigs, can shield the probiotics against unfavorable conditions in the GIT. In one study, it was shown that *L. plantarum* administered encapsulated with alginate and gelatin acted against the activity of *Salmonella* spp., *S. aureus*, *Listeria monocytogens*, and *E. coli* [118]. Probiotics were given to young pigs, which enhanced intestinal integrity, lengthened intestinal villi in the jejunum, revealed microbes that positively impacted the intestinal microbiota, and increased individual growth throughout the rearing cycle [119].

The sows become weak during pregnancy and lactation. *L. johnsonii* was isolated from the intestinal mucosa of healthy pigs, and subsequent in vitro tests confirmed that it had the capacity to show resistance to hydrochloric acid and bile salts and has inhibitory activity against pathogenic bacteria in co-culture [120]. Moreover, sows fed freeze-dried *L. johnsonii* exhibited a significant increase in body weight. These results indicated the positive impact of *L. johnsonii* on the production efficiency of sows. Moreover, it has been suggested that *L. johnsonii* is a promising substitute for the use of antibiotics in feed [120]. In another study, oral administration of *L. reuteri* showed high inhibitory activity against ETEC. This *Lactobacillus* strain improved the production ability of sows, boosted weaned piglets’ development, and decreased the incidence of diarrhea [121]. Additionally, *L. brevis* ZLB004 supplementation enhanced food digestion, gross weight, and gross energy [122]. Moreover, in this instance, the supplementation had an impact on the sow’s litter. Weaned pigs showed a lower mortality rate, and their weight growth was visible. In a study, it was shown that with oral feeding of *L. sobrius*, the number of *E. coli* in pigs’ feces decreased after they had been weaned [123].

Similar positive outcomes were observed when probiotic blends containing *L. acidophilus*, *L. delbrueckii*, *L. rhamnosus*, *L. plantarum*, and *Bifidobacterium bifidum* were administered to the sow. In this study, neither the sows nor the piglets experienced diarrhea during the course of the study [124]. Probiotics-supplemented piglets showed a rise in the concentration of acetic, propionic, and butyric acids in their feces [124]. Moreover, there was a significant change in the piglets’ intestinal environment. When given as a food supplement to sows alone, *L. plantarum* CAM6 improved body weight and decreased the occurrence of diarrhea in the progeny [125]. Moreover, sow milk’s nutritional values also increased. Providing a meal containing *L. reuteri* to weaned piglets improved fat digestion and absorption in the cecum and facilitated tryptophan metabolism [126]. Wang et al. [127] showed that the feeding of feed co-fermented with *B. subtilis* and *E. faecium* in lactating sows resulted in an improvement in the sow’s milk quality, and the weight of the piglets also increased. The modification of the intestinal microbiota was associated with a decrease in the frequency of constipation in sows and diarrhea in piglets. The piglets demonstrated superior immunity and performance [128]. In addition, the beneficial effects of *Bacillus coagulans* have been proven on the piglets’ physiology and physical condition [129]. The diet enriched with *B. coagulans* resulted in enhanced weight gain with a decreased feed ratio. *L. salivarius* feeding lowers the amount of *E. coli* in the cecum. In non-antibiotic breeding, the feeding of probiotics had a good impact on piglets’ intestinal integrity, immunity, and physical condition [117]. This option can be used as a substitute for antibiotic growth promoters. The potential benefits of live probiotics are summarized in Table 1.

## 6. Probiotics as Alternatives to Antibiotics and Zinc Oxide in Weaned Piglets 

Since post-weaning diarrhea (PWD) is a substantial barrier for pig production, antibiotics and zinc oxide (ZnO) are frequently used for the treatment and prevention of it. However, high levels of antibiotics and ZnO negatively impact human health and the environment by causing the emergence of antimicrobial-resistant bacteria and increasing the concentration of the heavy metal zinc in the soil. Previous studies have revealed that prophylactic use of dietary ZnO boosts other antibiotic-resistant genes, such as tetracycline and sulphonamide resistance genes of Gram-negative bacteria in weaning piglets [140,141]. Therefore, considering the negative impact of ZnO on public health and the environment, as well as the link between dietary zinc levels and the incidence of antimicrobial resistance, the EU decided to withdraw all veterinary products containing ZnO, which was enacted on 26 June 2017 [142]. This has led to an extensive search for nutritional and management practices that can help prevent PWD in the absence of pharmacological levels of ZnO and avoid a rise in the use of antibiotics. Among many other strategies, the use of probiotics as alternatives to antibiotics and zinc oxide in weaned piglets has gained attention in recent years. Several studies have shown that probiotics can improve growth performance, reduce the incidence of diarrhea, and enhance the immune response in weaned piglets. Dietary supplementation with *B. subtilis* reduced diarrhea severity, improved gut health, and alleviated systemic inflammation in orally infected weaned piglets with ETEC F18 [143]. Additionally, *B. licheniformis* decreased the amount of *E. coli* in GIT contents, encouraged the development of beneficial microbiota such as *Lactobacillus* in the ileum, and had a favorable effect on the pH of gut contents of weaned piglets [144]. It has been shown that supplementing *Bifidobacterium lactic* could lessen the severity of weaning diarrhea infected by rotavirus [145]. Moreover, the dietary administration of a multi-strain probiotic mixture of *B. longum* and *B. anomalies* could improve the *S.* Typhimurium challenge in weaned piglets [146]. *Enterococcus* (e.g., *E. faecium* NCIMB 10415) could also improve intestinal function by reducing the frequency of diarrhea in the piglets studied by Lodemann et al. [147]. Besides other probiotic strains, *Lactobacillus* plays an important role in improving the growth performance and intestinal health of weaned piglets. Dietary supplements of *L. plantarum* and *L. reuteri*, alone or in mixture, significantly decreased the incidence of diarrhea and fecal scores in piglets [148]. According to Rodriguez et al. [149] administering *L. rhamnosus* HN001 at the optimal dose to piglets reinforced the preventive effect of *B. longum* against *E. coli*. However, while probiotics offer potential benefits as alternatives to antibiotics and ZnO in weaned piglets, it is important to note that the effectiveness of probiotics as alternatives to antibiotics and zinc oxide may vary depending on the specific strains used, their dosage, and the management practices employed.

## 7. Cost–Benefit Analysis of Using Probiotics in the Swine Industry

The choice to use different kinds of probiotics depends on how much it will cost, how simple it will be, and whether it will improve production efficiency or boost total profits for the swine industry. Moreover, some other significant aspects of probiotic use are also considered, including strain and dose specificity, mode of action, and interactions with the host [150]. Based on empirical and literature studies, efforts and advances have been made to select and characterize probiotics for use in pigs. A variety of commercial probiotics for swine are currently available in the market, and small doses of these additives are frequently used. One study revealed that probiotics typically accounted for USD 13.65 billion in 2019 and are estimated to increase by 6.1% in 2027, depending on the manufacturer and the product’s active components [151]. In commercial pig farming, higher growth performance and feed efficiency are likely to result in lower production costs. Additionally, if the pigs can fend off disease and live until they are big enough to be marketed, the subsequent cost of treatment and overall production costs would be significantly decreased. It has been shown in a study that using probiotics as dietary supplements in pigs revealed a lower cost per kg of live pigs after accounting for the cost of antibiotic treatment [152]. In another study, the combination of a higher daily weight gain and a slight decrease in the feed conversion ratio linked to the addition of the probiotic may have contributed to the lower cost of production, even after accounting for the probiotic’s additional costs, with an estimated increase in income per pig, offering an economically viable alternative to increasing pig production [153]. A study by Rybarczyk et al. [154] using probiotics in pigs by feeding commercial EM^®^ probiotics may result in more benefits than the formulation cost. The study’s findings revealed that adding EM^®^ probiotics supplements to the piglet diet can improve growth performance, feed efficiency, and meat quality with economic efficiency. Including these commercial probiotics in pig starter and finisher diets was encouraging from an economic standpoint since treated groups produced more profit than the control group [154]. According to Kenny et al. [155], probiotics can fully demonstrate a positive response in commercial settings, particularly in an unhygienic environment where subclinical infections may occur. Probiotic feeding mostly improves immunity and prevents subclinical illnesses. Therefore, this circumstance would offset the economic costs of antibiotic therapies for the pigs by increasing their immune response. However, generally, probiotic effects are less extensive than those that antibiotics can produce. As a result, in some countries where antibiotic usage is not prohibited by legislation, applying probiotics may not be cost-effective. Moreover, the cost of probiotics may vary depending on the strain, dose, context, and host-related physiological parameters [156,157].

## 8. Safety Concerns Involving the Use of Live Probiotics

Despite the extensive use of probiotic supplements and their effectiveness in treating various disorders, some worries have been expressed regarding the unfavorable reactions of live probiotic strains, especially in children and adults suffering from severe diseases [28,29]. One of the main concerns with using live probiotics is that they could move from the intestine to systemic circulation and surrounding draining tissues, which could cause bacterial infections, particularly in infants and immunocompromised patients [158,159]. Other worries about using live probiotics comprise the possibility of probiotic strains transmitting antibacterial resistance genes via gene transfer in the human GIT [160], the existence of harmful metabolic actions, and exciting immunological activation in vulnerable individuals [161]. Before the product is marketed, a thorough examination of the probiotic strains should be carried out, including genomic strain characterization to ensure the lack of resistance determinants, among other things [162]. A recent study revealed that food-borne *Lactobacillus* could transmit antibiotic resistance features to food pathogens, raising concerns regarding their use as probiotics or dietary supplements [163]. The use of live probiotic bacteria may generate stable colonies in neonates, stopping the regular colonization of other bacteria or the typical microbiota in the intestine and therefore suppressing the normal development of the immune system [164]. Short-term probiotic therapy in combination with antibiotics was found to be safe and beneficial in the case of a non-immunocompromised individuals or patients who are not seriously weakened, leading to the conclusion that susceptible patients must be informed about the possible advantages and hazards of probiotics [165]. Among many other concerns, bacterial sepsis and impaired intestinal barrier function of the immunocompromised and critically ill patient should be taken into consideration while taking probiotics [19]. In the case of using probiotics in neonates, the parents should be fully aware of the potential risk of probiotics [166]. Moreover, there are some reports describing dangerous infections such as endocarditis, septicemia, abscesses, and meningitis in patients treated with *Bacillus subtilis*, *Bifidobacteria*, and *Lactobacillus*, mainly in children and other immunocompromised patients [158,167]. Therefore, to avoid these discrepancies in using live probiotics, the use of postbiotics is gaining popularity day by day.

## 9. Postbiotics and Their Effects on Immunomodulation

To overcome the potential risks, using non-viable microorganisms or their cell extracts is now of great interest. The killed bacteria can still maintain adequate immunomodulating properties because cellular components and other accessory materials are inside the cells [168]. It is important to note that different methods of killing bacteria may have different effects on the structure and composition of the bacteria, which can affect their ability to interact with the immune system and exert immunomodulatory effects (Figure 1). Therefore, the choice of method for killing bacteria for use as immunomodulating agents should be carefully considered based on the specific bacterial strain and intended use. Probiotic bacteria can be killed by using various methods. The methods include the following.

### 9.1. Heat Treatment

Heat treatment is one of the efficient ways to kill bacteria as in high temperatures bacteria can easily die. This involves subjecting the bacteria to high temperatures for a certain period of time. This method is commonly used to prepare killed bacteria for use as immunomodulating and/or immunoprotective agents. Heat treatment can be done by autoclaving or boiling the bacterial culture. Previous studies showed that preparations comprising killed bacterial cells and their metabolites could exert biological responses, in many cases more than live cells [46]. Heat-killed probiotics showed competition for adhesion sites against several enteropathogens in the human colon carcinoma (Caco)-2 experimental model [169]. In terms of their capacity to compete for adhesion on gut epithelial cells, the promising potentiality of heat-inactivated probiotics may hint at the ability to fight against diarrhea and food-borne pathogenic infections. Furthermore, several heat-killed *Lactobacillus* probiotics have shown immunomodulatory (ability to enhance anti-inflammatory cytokines such as IL-6 and IL-10 and to suppress proinflammatory cytokines viz. IL-β and TNF-α) and antioxidative effects in in vitro and in vivo experimental models [42,170]. Heat-treated inactivated bacteria can provide bacterial constituents having key immunomodulatory or immunoprotective actions and preventive activities against many pathogens. Bacterial compounds such EPSs, peptidoglycans, and lipoteichoic acids are involved to show these types of properties in the preparation of heat-inactivated bacteria [171]. 

### 9.2. UV Irradiation

UV light can be used to kill bacteria by disrupting their DNA. UV-killed probiotics are non-viable probiotic microorganisms that have been inactivated by exposure to UV radiation. While live probiotics are known to confer various health benefits, such as promoting gut health and improving immunity, there is increasing interest in the potential benefits of non-viable probiotics, particularly in the area of immunomodulation. Research has suggested that UV-killed probiotics can have immunomodulatory effects, such as stimulating the immune system and regulating the immune response [172]. These effects are thought to be due to the presence of various components within the probiotic cell wall, such as peptidoglycans and lipoteichoic acids, which can activate immune cells. Several studies have demonstrated the potential immunomodulatory effects of UV-killed probiotics. For example, one study found that UV-killed *Lactobacillus casei* 32G modulated the immune response in mice by increasing the production of cytokines and enhancing the activity of immune cells [173]. Another study suggested that UV-killed probiotics improved the immune response in elderly individuals by increasing the production of antibodies [174].

### 9.3. Sonication

Sonication is a process that uses high-frequency sound waves to disrupt the cell walls of probiotic bacteria, resulting in non-viable or inactivated bacteria that may still have immunomodulatory effects. Studies have shown that sonicated probiotics have beneficial effects on various aspects of immune function, including increasing the production of certain immune cells, improving the response to infections, and reducing inflammation [175,176]. One study found that sonicated *Lactobacillus acidophilus* increased the production of the anti-inflammatory cytokine IL-10 in human immune cells [35].

## 10. Beneficial Effects of Inactivated Probiotics on Pigs

While live and active forms of LAB are commonly used as probiotics in humans and animals, inactivated LAB have also gained some attention in research and certain applications. In the context of pigs, inactivated *Lactobacillus* has been investigated for its potential benefits in pig farming. Some studies have explored its use as a feed additive to improve swine health and performance. Inactivated *Lactobacillus* may have immunomodulatory properties, and its inclusion in pig feed has been suggested to enhance the immune response, reduce the incidence of diarrhea, and improve growth performance in piglets. Previous studies conducted on weaning piglets found that the inclusion of inactivated *Lactobacillus* in their feed improved growth performance, reduced diarrhea occurrence, and positively influenced the composition of the gut microbiota [122,177,178]. One study investigated the effects of heat-inactivated *Lactobacillus reuteri* I5007 on pregnant sows and reported improved litter performance and reduced occurrence of post-weaning diarrhea in piglets [121]. Good et al. [179] showed that UV-inactivated *L. rhamnosus* HN001 decreased the severity of necrotizing enterocolitis in preterm piglets. While these studies suggested potential benefits, further research is still needed to fully understand the efficacy, dosage, and optimal application of inactivated *Lactobacillus* in pig farming. Additionally, it is worth considering that the specific strain of *Lactobacillus*, the method of inactivation, and other factors can influence the outcomes. The overall benefits of using inactivated probiotics are summarized in Table 2.

## 11. Conclusions

The use of antibiotics to treat several diseases in both humans and animals was the greatest discovery in the history of therapeutics. Unfortunately, the overuse and/or abuse of antibiotics has led to the emergence of antibiotic-resistant bacteria in both human and veterinary medicine. Large-scale pig breeding aims to increase productivity and decrease pig herd mortality. Probiotics in their live and inactivated forms can be alternatively used in pigs for the balance restoration of the gut microbiota, immunomodulation or immunoprotection, pathogen protection, and maintenance of intestinal barrier integrity. They are promising for use in pigs to improve feed efficiency, nutrient digestibility, gut integrity, growth performance, and immunity, as well as to reduce diarrhea and bacterial infections. Therefore, live and inactivated LAB can be used in the growing functional feed sector for pigs.

## Figures and Tables

**Table 1:** Overview of the use of live probiotics in pigs.

Probiotics	Effects	References
*Lactobacillus* *delbrucei* TUAA4408L	Boost immunity and modulate intestinal microbiota; improve growth performance and meat quality	[130]
*L. acidophilus*	Improve feed efficiency	[131]
*Lactobacillus frumenti*	Decrease incidence of diarrhea	[132]
*L. reuteri*	Improve feed intake and weight gain	[133]
*L reuteri and Bacillus licheniformis*	Increase nutrient digestibility	[126]
*L. rhamnosus* GG	Improve gut integrity	[134]
*L. fermentum* ZLP001	Enhance catalase and superoxide dismutase	[135]
*L. salivarius* 160	Elevate the levels of beneficial bacteria and decrease harmful bacteria	[117]
*L. plantarum* VTT E-78076	Improve growth performance and gastrointestinal ecology	[124]
*L. brevis* ZLB004	Improve intestinal microbiota balance, immunity and growth performance	[122]
*L. jensenii* TL2937	Modulate porcine mononuclear phagocytic activity	[136]
*L. plantarum* SC01	Inhibit the growth of *Salmonella*, *E. coli*, and *S. aureus*	[118]
*L. casei*	Improve immune functions and gut microbiota balance	[137]
*L. reuteri* I5007	Improve intestinal infection defense	[121]
*L. johnsenii* XS4	Improve reproductive performance and immunological index	[120]
*L. plantarum* CAM6	Develop milk quality	[125]
*L. amylovorus* DSM16698	Enhance microbial community structure and metabolite production	[138]
*L. salivarius* B1	Develop intestinal mucosal immunity	[128]
*L. plantarum*	Promote intestinal barrier functions	[139]
*L. sobrius*	Improve ETEC infections	[123]

**Table 2:** Overview of the use of inactivated probiotics in pigs.

Probiotics	Mode of Inactivation	Effects	References
*L. planturum* L-137	Heat	Improve growth performance, intestinal morphology, and immune gene expression	[180]
*Ligitolactobacillus salivarius* 189	Heat	Modulate body weight and gut microbiota composition	[181]
*L. rhamnosus*	Heat	Develop growth performance and immune response	[177]
*L. farciminis* 3699	Heat	Decrease the incidence of dysentery	[182]
*L. plnatarum*	Heat	Elongate villi	[183]
*L. rhamnosus* MA27/6B *and L. acidophilus* MA27/6R	Heat	Promote growth performance	[184]
*L. rhamnosus* CRL1505	Heat	Boost immunity	[178]
*L. reuteri* I5007	Heat	Improve intestinal infection defense	[121]
*Limosilactobacillus fermentum*	Heat	Prevent post-weaning diarrhea and improve growth efficiency	[185]
*L. rhamnosus* HN001	UV	Attenuate necrotizing enterocolitis	[179]

## Data Availability

Not applicable.

## Abbreviations

LAB	Lactic acid bacteria
GIT	Gastrointestinal tract
EPS	Exopolysaccharides
UV	Ultraviolet
TLR	Toll-like receptor
SCFA	Short-chain fatty acid
BS	Biosurfactants
IL	Interleukin
INF	Interferon
TNF	Tumor necrotic factor
S-layer	Surface layer
PBMC	Peripheral blood mononuclear cell
Treg	Regulatory T cell
NK	Natural killer
sIgA	Secretory immunoglobulin A
dIgA	Dimeric Immunoglobulin A
Caco-2	Colon carcinoma-2
ETEC	Enterotoxigenic *E. coli*
